# Bridge symptoms of depression and anxiety among older adults in China: a longitudinal network comparison by living arrangements

**DOI:** 10.3389/fpsyt.2025.1681404

**Published:** 2025-10-17

**Authors:** Hao Xu, Xiaowen Li, Cui Liu

**Affiliations:** ^1^ School of Physical Education, Sichuan Institute of Industrial Technology, Deyang, China; ^2^ Science and Technology Department, Sichuan Institute of Industrial Technology, Deyang, China

**Keywords:** mental health in older adults, depression and anxiety, living arrangement, symptom network analysis, propensity score matching (PSM), network comparison test (NCT)

## Abstract

**Background:**

Comorbidity of depression and anxiety is highly prevalent among older adults, yet longitudinal evidence on how different living arrangements shape the interactions between these symptoms remains scarce.

**Methods:**

Data were drawn from the 2011, 2014, and 2018 waves of the Chinese Longitudinal Healthy Longevity Survey (CLHLS). Participants aged ≥60 years who completed both the CES-D-10 and GAD-7 were included. After 1:1 propensity score matching (PSM) on key demographic variables, the final analytic sample comprised 834 older adults. Bayesian Gaussian Graphical Models were applied to construct contemporaneous and lag-1 temporal networks. Bridge edges linking depressive and anxiety clusters were identified, and group differences were examined using the Network Comparison Test (NCT).

**Results:**

The mean age of the sample was 84.5 years; 61.7% were female, and 84.5% held rural hukou. In the overall sample, the strongest bridge edge was between CESD10 (poor sleep quality) and GAD1 (feeling nervous, anxious, or on edge) (*r* = 0.105). Subgroup analyses revealed distinct bridge-symptom pathways: a “sleep–anxiety” pathway in those living alone (CESD10–GAD1, *r* = 0.161) and a “tension–worry” pathway in those living with family (CESD6–GAD6, *r* = 0.130). The NCT indicated no significant difference in global network strength (Δ = 0.131, *p* = 0.706), but five cross-cluster edges differed significantly between groups (*p* < 0.05).

**Conclusions:**

Living arrangements shape the bridge-symptom mechanisms linking depressive and anxiety symptoms in later life. Interventions for older adults living alone should prioritize improving sleep, whereas those for older adults living with family should emphasize emotional regulation and family support. These findings provide longitudinal, network-based evidence on context-specific comorbidity mechanisms and offer empirical guidance for tailored public health and clinical interventions.

## 1 Introduction

As the global population ages, the mental health of older adults has become a prominent public health concern. The World Health Organization (WHO) projects that by 2050, the number of individuals aged ≥60 years will exceed 2 billion, and depression and anxiety will be major contributors to declines in quality of life and functional capacity (1). Evidence indicates that the comorbidity of depression and anxiety in later life can reach 45.7%, markedly increasing the risks of limitations in activities of daily living, disability, and even mortality, thereby imposing a substantial burden on healthcare systems (2). In China’s primary care settings, approximately one in five older patients meets the threshold for depressive symptoms (3), and a comparable proportion presents with clinically significant anxiety (4). Epidemiological studies further demonstrate that living arrangements are closely linked to these mental health outcomes; in particular, living alone is associated with higher risks of depression and anxiety, and this association is partly transmitted through lifestyle pathways (e.g., insufficient sleep and unhealthy diet), underscoring modifiable intervention targets (5, 6). At the same time, this association exhibits contextual heterogeneity: in certain family contexts, co-residence with relatives is not uniformly protective, as caregiving burden, intergenerational conflict, and household crowding may exacerbate psychological distress (7). Accordingly, living arrangement is not merely a demographic attribute; via modifiable psychosocial and behavioral mechanisms, it functions as a important structural determinant for understanding mental health disparities among older adults.

Researchers have sought to examine this phenomenon from the perspective of varying living arrangements. For example, Fang et al. (8), using data from the 2020 China Health and Retirement Longitudinal Study (CHARLS), reported that depressive symptoms were significantly more prevalent among older adults living alone compared with those living with others and that participation in social activities and satisfaction with intergenerational relationships partially mediated this association (8). Gao et al. (9) focused on rural older adults in Northwest China and found that the risk of depression was particularly high among those living alone, especially when social support was lacking and community resources were insufficient—conditions that also intensified comorbid anxiety symptoms (9). These findings not only validate the strong link between solitary living and psychological distress, but also suggest the mediating role of the “social support–mental health” pathway. International studies offer additional insights. Honjo et al. (10), drawing on longitudinal data from the Japanese JAGES project, found that social cohesion significantly moderated the relationship between living arrangement and depression: in communities with high social cohesion, the risk of depression among those living alone was reduced, whereas in socially fragmented communities, the adverse effects of solitary living were amplified (10). Park et al. (11), in a Korean context, further showed that living alone not only increased depressive symptoms but was also bidirectionally associated with cognitive decline, highlighting a vicious cycle between psychological distress and functional deterioration (11).

Moreover, existing studies suggest that living with family members is not necessarily a psychologically protective factor. Jia et al. (12), based on longitudinal analyses of the CLHLS, argued that the alignment between actual living arrangements, individual preferences, and perceived fit is a key determinant of depressive symptoms among older adults. When residential preferences and reality are misaligned—such as older adults desiring to live with children but being forced to live alone, or preferring solitude but compelled to live with family—psychological distress tends to rise significantly (12). Zhang et al. (7) further emphasized that, within the Chinese cultural context, different living arrangements (e.g., living alone, living with a spouse, or living with both spouse and children) have complex implications for mental health. In some cases, older adults living with family report higher levels of psychological distress, potentially due to intergenerational conflict, hidden caregiving pressure, and unequal distribution of family resources (7). While these studies provide foundational insights into the relationship between living arrangement and mental health, most focus on macro-level risk assessments and social determinants, with limited attention to structural differences at the symptom level and mechanisms of cross-symptom interactions.

To address this gap, symptom network analysis offers a new paradigm for mental health research. This approach conceptualizes psychopathology as a complex network of interacting symptoms, enabling the identification of highly influential central symptoms as well as bridge symptoms that transmit distress across diagnostic boundaries (13). Prior work shows that network analysis helps elucidate the structure of comorbidity and highlights the pivotal role of bridge symptoms in the mutual influence between different mental disorders (14). The “causal systems theory” further proposes that bridge symptoms function as conduits through which disturbance propagates between symptom clusters (15). In the depression–anxiety domain, symptoms such as sleep disturbance and excessive worry often occupy key network positions and serve as cross-cluster connectors (16); in China, Chen et al. were the first to construct an anxiety–depression symptom network among older adults living alone and identified sleep disturbance and anxiety as highly active bridges (17). However, most studies focus on a single subgroup (e.g., living alone), and systematic comparisons across different living arrangements (living alone vs. living with family) remain scarce, leaving the implications of context-specific symptom connectivity for intervention unclear. Building on this evidence and gap, from a methodological perspective, traditional latent-variable models treat items as manifestations of an underlying construct and are limited in capturing item-level heterogeneity and dynamic symptom–symptom coupling; by contrast, psychological network analysis aligns better with our aim to reveal cross-cluster linkages and contextual differences (18). Moreover, methodological work raises concerns about the reliability and cross-sample replicability of certain centrality indices (e.g., betweenness, closeness) in psychopathology networks, suggesting greater emphasis on strength and bridge metrics in practice (18, 19). Accordingly, we do not prioritize “central symptoms” as primary intervention targets; instead, we focus on bridge symptoms with stronger theoretical coherence and practical actionability, and we test whether bridge pathways differ systematically by living arrangement.

Against the above theoretical and methodological backdrop, the extant literature still exhibits notable shortcomings in study design and methodology. First, most investigations have employed cross-sectional designs, which are inherently unable to capture the temporal dynamics and causal directionality of symptom–symptom interactions (20). Second, conventional studies often rely on sum scores, aggregating all items into a single total and thereby overlooking symptom-level heterogeneity and network properties; this practice restricts the identification of precise intervention targets (21). While some research has constructed depression–anxiety symptom networks for specific subpopulations—such as older adults living alone (3)—comparative analyses of network structures across different living arrangements remain scarce. As a result, it remains unclear which symptoms serve as bridges in distinct residential contexts and how these symptoms facilitate the cross-group propagation of psychological distress. Bringmann et al. (19) highlighted that future research in network psychopathology should integrate longitudinal designs and contextual comparisons to elucidate dynamic connectivity patterns shaped by varying social structures (19). The present study aligns with this direction. By incorporating propensity score matching (PSM) to control for demographic confounders and employing the Network Comparison Test (NCT) to evaluate group-level differences in network structure, we provide a robust empirical foundation for symptom-level cross-group comparison. Moreover, this methodological integration offers valuable contributions from a Chinese population to the global advancement of network-based approaches in psychopathology research (22).

This study offers contributions at theoretical, methodological, and practical levels. Theoretically, by incorporating living arrangements (i.e., living alone vs. living with family) into symptom network analysis, the study addresses a significant gap in the literature concerning “social context–symptom connectivity” and deepens our understanding of depression–anxiety comorbidity among older adults (23). Methodologically, the integration of PSM and the NCT enhances the comparability and robustness of group-level network structure comparisons (22). Practically, the study seeks to identify key nodes within depression–anxiety symptom networks under different living arrangements, thereby offering theoretical rationale and empirical guidance for subgroup-specific interventions and community mental health services (16). Collectively, the findings expand the application of network analysis in geriatric mental health research and offer new insights for context-sensitive public health interventions.

## 2 Methods

### 2.1 Study design and data source

This study conducted a longitudinal network analysis based on data from the 2011, 2014, and 2018 waves of the Chinese Longitudinal Healthy Longevity Survey (CLHLS). The CLHLS covers 23 provinces, municipalities, and autonomous regions across China and is implemented by the Center for Healthy Aging and Development Studies/National School of Development at Peking University. It is one of the most representative longitudinal cohort studies of older adults in China. The CLHLS has received ethical approval from the Institutional Review Board of Peking University (IRB approval number: IRB0000105213074). All participants (or their legal proxies) provided written informed consent, and the study procedures comply with the ethical standards outlined in the Declaration of Helsinki. Focusing on individuals aged ≥60 years, the present study integrated data from three waves to examine the network structure and temporal dynamics of depressive and anxiety symptoms. In addition, the study compared symptom network characteristics between older adults living alone and those living with family members.

### 2.2 Sample and variable description

#### 2.2.1 Living arrangement

Based on the survey item “A5-1: Who do you currently live with?”, living arrangement was categorized into two groups: living with family members (including spouse, children, or live-in caregivers) and living alone. Due to the very small number of cases residing in institutional care, such individuals were excluded from the analysis.

#### 2.2.2 Mental health variables

Depression was measured using the 10-item short form of the Center for Epidemiologic Studies Depression Scale (CES-D-10), with items rated on a 5-point Likert scale (1 = always, 5 = never). Items were coded such that higher scores indicate greater levels of depressive symptoms. The CES-D-10 has been widely validated among older adult populations in China, demonstrating good reliability and validity (24, 25). In the present study, the Cronbach’s alpha for the CES-D-10 was 0.80, indicating satisfactory internal consistency.

Anxiety was assessed using seven items derived from the Generalized Anxiety Disorder Scale (GAD-7), adapted within the CLHLS framework. Responses were rated on a 4-point scale (0 = not at all, 3 = nearly every day), with higher scores reflecting more severe anxiety symptoms. The GAD-7 has been widely used and validated in older adult populations in China (26), and has shown strong psychometric properties within the CLHLS dataset (27). In this study, the Cronbach’s alpha coefficient for the anxiety scale was 0.83, indicating high internal consistency.

#### 2.2.3 Control variables

To adjust for potential confounding factors, the following covariates were included: gender, age, hukou type (urban/rural), self-rated health (1 = very good to 5 = very poor), average sleep duration (hours per night), limitations in activities of daily living (1 = severely limited, 2 = somewhat limited, 3 = not limited), years of education, marital status, and number of children. For categorical variables, missing values (coded as 8/9) were uniformly recoded as “unknown.” For continuous variables such as sleep duration, missing values were imputed using the median.

### 2.3 Propensity Score Matching

**Figure 1:**
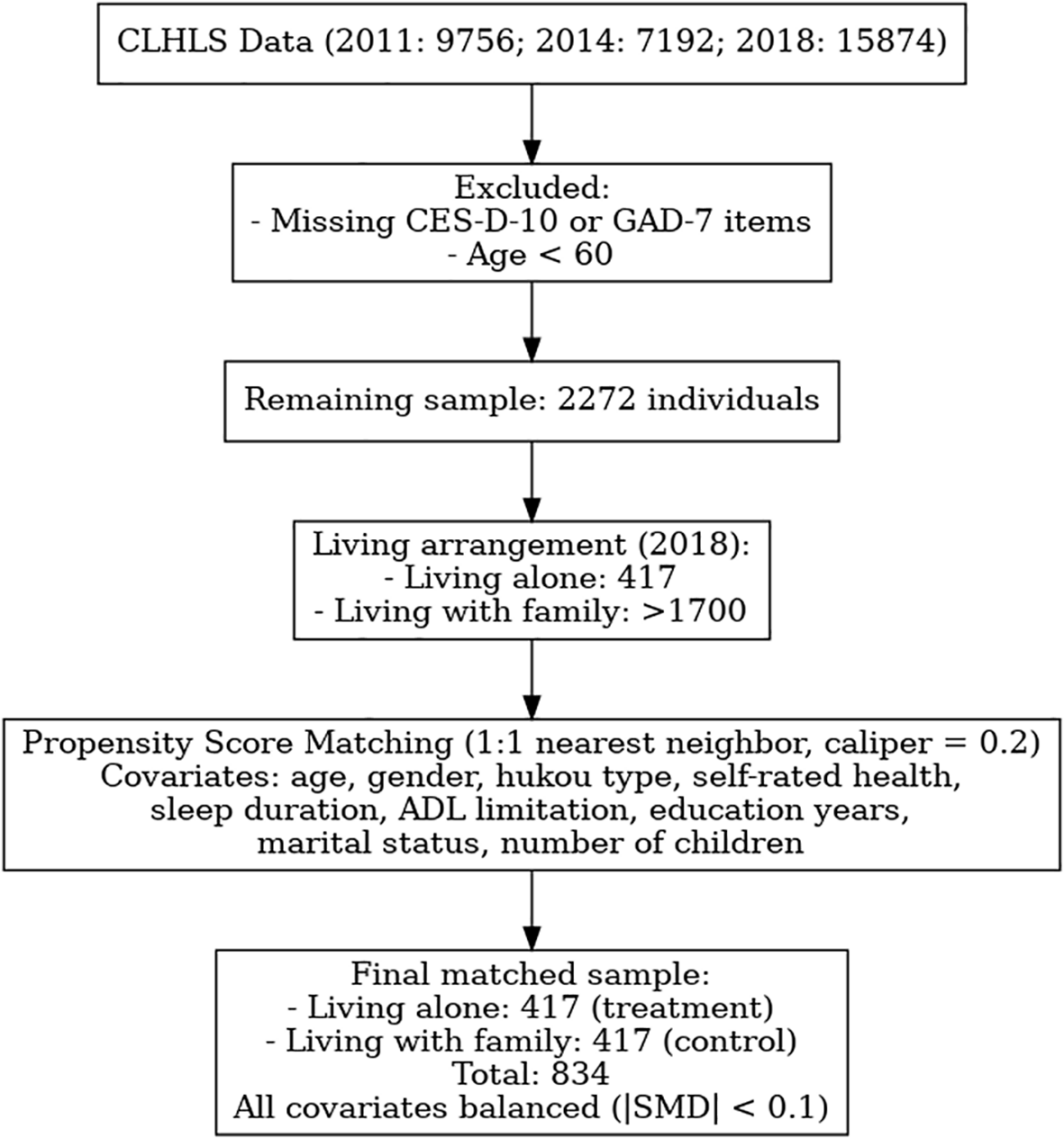
Flowchart of participant selection and propensity score matching.

To reduce baseline differences between groups with different living arrangements, the present study employed Propensity Score Matching (PSM) prior to network analysis. Data were drawn from the 2011, 2014, and 2018 waves of the CLHLS, including only those cases with complete responses to the CES-D-10 and GAD-7 items and limited to individuals aged 60 years or older. After initial screening, a total of 2,272 valid cases were retained, among which 417 participants were living alone—a significantly smaller number compared to over 1,700 living with family members. The flowchart of participant inclusion, exclusion, and propensity score matching is shown in **Figure 1**.

**Figure 2:**
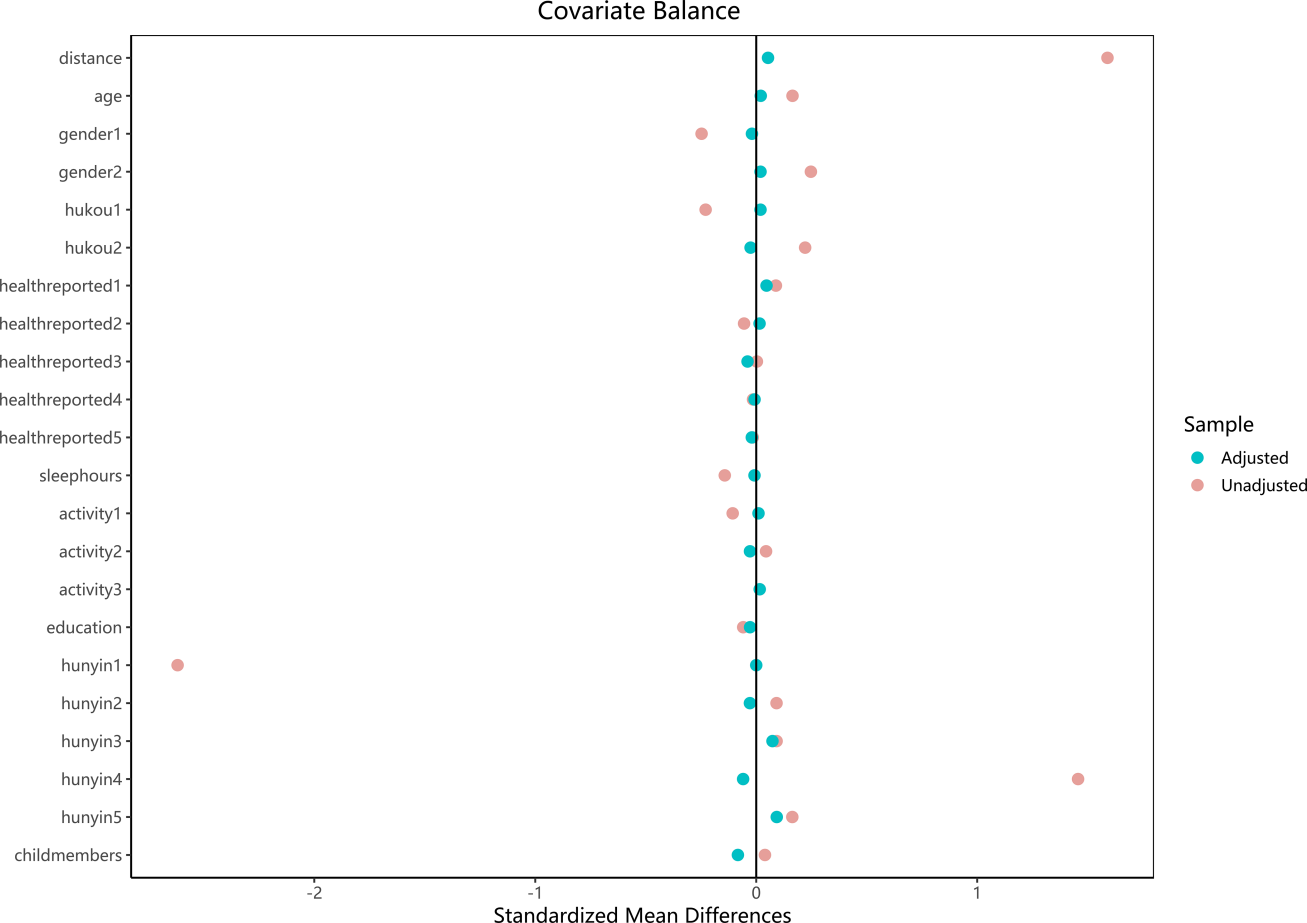
Covariate balance before and after matching. Standardized mean differences (SMDs) are plotted for all covariates. Red = unadjusted, Blue = adjusted.

Accordingly, the 417 older adults living alone were designated as the treatment group, and 1:1 nearest neighbor matching without replacement was conducted against the larger comparison group (living with family members), using a caliper width of 0.2. Covariates used for matching included: age, gender, hukou type (urban/rural), self-rated health, average sleep duration, limitations in activities of daily living, years of education, marital status, and number of children. Following matching, a total of 834 participants (417 in each group) were included in the final analysis. The standardized mean differences (SMDs) for all covariates after matching were close to zero (|SMD| < 0.1), indicating a high degree of balance between the two groups (see **Figure 2**). Detailed matching statistics are provided in **Supplementary Material S1**.

### 2.4 Network analysis

We conducted a network analysis in three steps to examine the structure and temporal dynamics of depressive and anxiety symptoms among older adults. First, step, two types of networks were estimated using the matched full sample (n = 834): (1) a contemporaneous network, capturing associations among symptoms at the same time point, and (2) a temporal network, capturing predictive associations between symptoms across different waves with a lag of one time point. Second, participants were stratified by living arrangement (living alone vs. living with family), and contemporaneous and temporal networks were estimated separately for each group. This allowed for direct comparison of symptom connectivity patterns and their evolution over time under different social contexts. All networks were estimated using the Bayesian Gaussian Graphical Models (BGGM) package in R, based on Bayesian posterior sampling (2,000 iterations). Visualization was performed using the qgraph package. Third, we applied the Network Comparison Test (NCT) to statistically assess differences between the two groups. Specifically, we tested for differences in (1) network structure (structure invariance), (2) overall connectivity strength (global strength invariance), and (3) individual edge weights (edge invariance). The NCT was performed with 1,000 permutations, and significance was set at *p* < 0.05.

## 3 Results

### 3.1 Sample characteristics

The final analytic sample consisted of 834 older adults, including 417 individuals living alone and 417 matched individuals living with family. As shown in **Table 1**, the mean age of the participants was 84.49 years (SD = 7.54), and 61.7% were female. A majority (84.5%) held rural household registration status. Educational attainment was generally low, with an average of 1.24 years of formal schooling (SD = 2.84). In terms of perceived health, 37.5% of respondents rated their health as “fair,” and 36.8% as “good.” Notably, 70.3% reported no limitations in activities of daily living. Regarding marital status, the vast majority (90.0%) were widowed, while only 4.7% were currently married and living with a spouse.

**Table 1:** Sample characteristics.

Variable	Mean ± SD/n (%)
Age	84.49 ± 7.54
Sleep hours (per night)	6.96 ± 2.23
Education (years)	1.24 ± 2.84
Number of children	2.32 ± 2.67
Living arrangement	With household member: 417 (50.0%); Alone: 417 (50%)
Gender	Male: 319 (38.3%); Female: 515 (61.7%)
Hukou	Urban: 128 (15.4%); Rural: 705 (84.5%)
Self-rated health	Very good: 113 (13.6%); Good: 307 (36.8%); So-so: 313 (37.5%); Bad: 90 (10.8%); Very bad: 11 (1.3%)
Activity limitation	Strongly limited: 55 (6.6%); Limited: 189 (22.7%); Not limited: 586 (70.3%)
Marital status	Married & living with spouse: 39 (4.7%); Separated: 18 (2.2%); Divorced: (1.2%); Widowed: 751 (90.0%); Never married: 16 (1.9%)

### 3.2 Contemporaneous and temporal networks in the total sample

**Figure 3:**
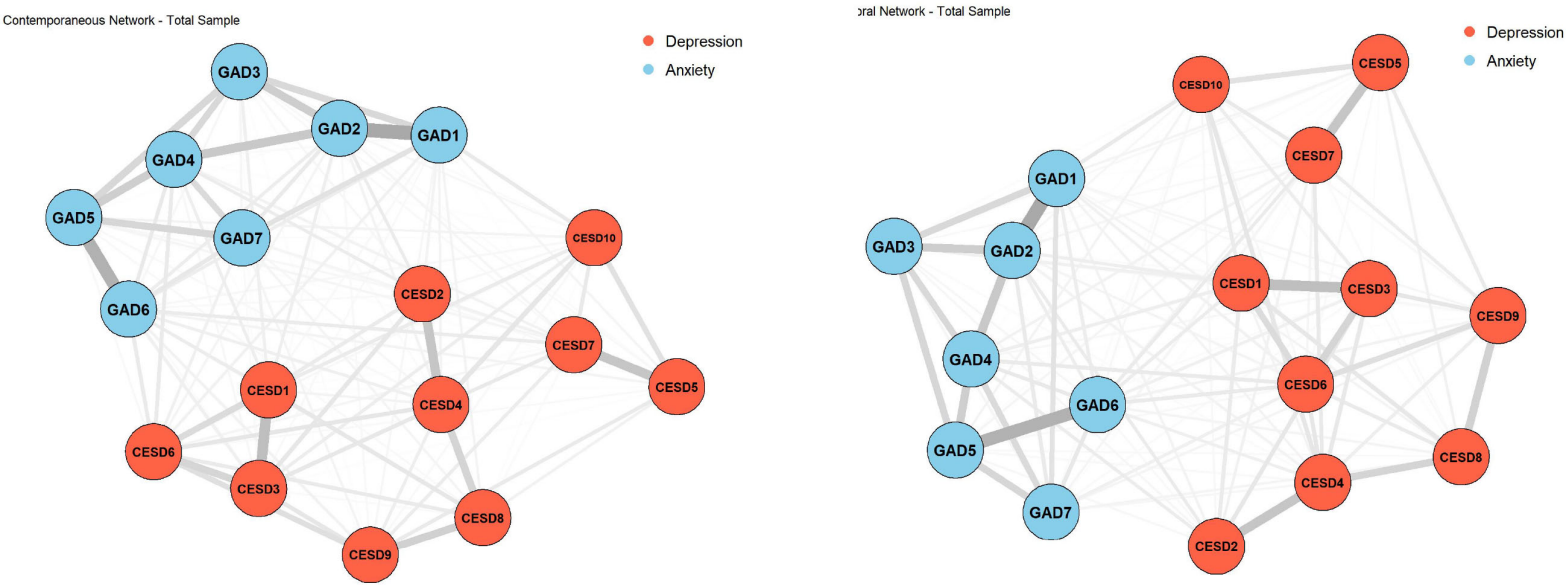
Contemporaneous and temporal symptom networks of depression and anxiety in the total sample. Edge thickness represents the magnitude (absolute value) of the estimated edge weight: partial correlations for contemporaneous networks and standardized lag-1 predictive associations for temporal networks. Node labels (CESD1–10, GAD1–7) represent individual items from the CES-D and GAD-7 scales, respectively. See **Appendix A** for complete item descriptions.

Based on three-wave longitudinal data from 834 older adults (collected in 2011, 2014, and 2018), we estimated both a contemporaneous network and a temporal network using Bayesian Gaussian Graphical Models (BGGM). These networks revealed the static structure and dynamic interactions of depressive and anxiety symptoms over time (see **Figure 3**; 95% credible intervals are reported in **Supplementary Material S2**).

In the contemporaneous network, depressive and anxiety symptoms formed a highly interconnected structure. Within the depression cluster, several symptom pairs demonstrated particularly strong associations, including CESD1 (being bothered by trivial matters) and CESD3 (feeling depressed or sad) (*r* = 0.316), CESD2 (difficulty concentrating) and CESD4 (feeling useless or having difficulty doing things due to aging) (*r* = 0.279), and CESD5 (feeling anxious about the future) and CESD8 (feeling lonely) (*r* = 0.278), which together constituted a central symptom chain. A similarly cohesive pattern was observed within the anxiety domain, with GAD1 (feeling nervous, anxious, or on edge) and GAD2 (not being able to stop or control worrying) (*r* = 0.423), as well as GAD5 (being so restless that it is hard to sit still) and GAD6 (becoming easily annoyed or irritable) (*r* = 0.380), emerging as the most strongly connected pairs. These results highlight the co-occurrence of anxious arousal, excessive worry, and emotional reactivity as a core component of the anxiety network.

Cross-cluster associations were also prominent, revealing bridge mechanisms between depressive and anxiety symptoms. The strongest bridge edge was found between CESD10 (poor sleep quality) and GAD1 (*r* = 0.105), suggesting a mutually reinforcing relationship between sleep disturbance and anxiety experiences at the same time point. Additional links such as CESD6 (feeling nervous or afraid) with GAD4 (difficulty relaxing) (*r* = –0.099) and GAD6 (*r* = 0.098) indicate bidirectional interactions between physical tension and emotional irritability. Additionally, CESD7 (feeling as happy as one was when younger) and GAD6 (feeling nervous or afraid) showed a negative association (*r* = −0.092).

The temporal network further revealed longitudinal predictive relationships among symptoms. Core depressive symptoms such as CESD1–CESD3 and core anxiety symptoms such as GAD1–GAD2 remained consistently associated across the three survey waves, indicating temporal stability. Cross-symptom effects were also evident: CESD10 had the most pronounced longitudinal predictive effect on GAD1 (*r* = 0.105), indicating that sleep problems and physical tension not only co-occur with anxiety at the same time point but are also positively associated with subsequent anxiety levels. In addition, the effects of CESD6 on GAD4 (*r* = −0.099) and GAD7 (*r* = −0.069) persisted over time and were negative in direction.

In summary, the network analysis of the total sample revealed a highly interconnected and structurally stable comorbidity network of depression and anxiety. Both within-cluster cohesion and cross-cluster bridge effects were identified. In particular, the persistent connection between poor sleep and anxiety, as well as the bidirectional interaction between tension and irritability, offer key insights into the mechanisms of comorbidity and provide a foundation for subsequent subgroup network comparisons. However, whether the network symptom structure changes between live alone and live with family will be further verified by subsequent group analysis.

### 3.3 Group-level network comparison (living alone vs. living with family)

**Figure 4:**
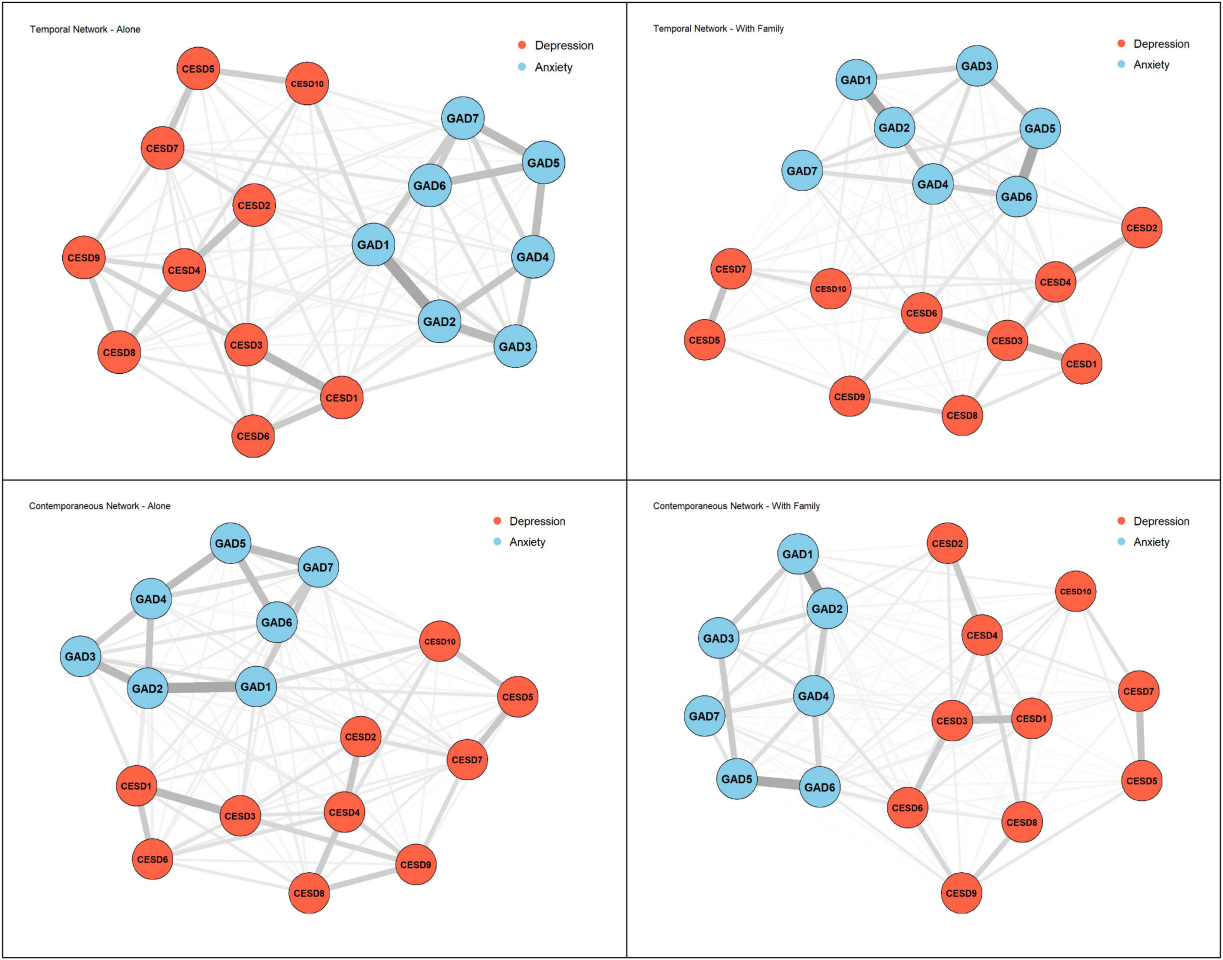
Group comparison of contemporaneous and temporal networks between older adults living alone and those living with family. Edge thickness represents the magnitude (absolute value) of the estimated edge weight: partial correlations for contemporaneous networks and standardized lag-1 predictive associations for temporal networks. Node labels (CESD1–10, GAD1–7) represent individual items from the CES-D and GAD-7 scales, respectively. See **Appendix A** for complete item descriptions.

Based on the propensity-score–matched subsamples, we constructed both contemporaneous and temporal networks for each group to compare the structural characteristics and dynamic interconnections of depression–anxiety symptoms across different living arrangements (see **Figure 4**).

In the contemporaneous networks, both groups of older adults exhibited distinct symptom clusters, with depressive symptoms (CESD1–10) and anxiety symptoms (GAD1–7) forming tightly connected sub-networks. Among the anxiety symptoms, GAD1 (feeling nervous, anxious, or on edge)–GAD2 (not being able to stop or control worrying) represented the strongest within-cluster connection in both groups (living alone: *r* = 0.392; living with family: *r* = 0.444), indicating that excessive worry, consistent with the total-sample analysis, constitutes a stable core of the anxiety network. However, GAD5 (being so restless that it is hard to sit still)–GAD6 (becoming easily annoyed or irritable) was particularly strong in the living-with-family group (*r* = 0.432) but weaker in the living-alone group (*r* = 0.288), suggesting that co-residence may increase the co-occurrence of irritability-related symptoms. In addition, within the depression cluster, older adults living alone showed a stronger connection between CESD1 (being bothered by trivial matters)–CESD3 (feeling depressed or sad) (*r* = 0.312), while the corresponding edge was comparably strong in the living-with-family group (*r* = 0.325), indicating that their depressive experiences are more centered on negative affect.

Cross-symptom connections revealed structural differences between the two networks and their divergence from the total-sample analysis. Among older adults living alone, CESD10 (poor sleep quality)–GAD1 (feeling nervous, anxious, or on edge) was the strongest cross-cluster edge (*r* = 0.161), whereas the corresponding edge in the living-with-family group was weaker (*r* = 0.076), consistent with the total-sample findings. The next strongest cross-cluster connections were CESD1 (being bothered by trivial matters)–GAD3 (bothered by trivial matters–excessive worry; *r* = –0.121) and CESD7 (feeling as happy as one was when younger)–GAD6 (becoming easily annoyed or irritable; *r* = –0.115). This pattern indicates that sleep disturbance and diminished positive affect constitute key pathways linking depression and anxiety among the solitary elderly. In contrast, for older adults living with family, the most prominent cross-cluster connections were CESD6 (feeling nervous or afraid)–GAD6 (becoming easily annoyed or irritable; *r* = 0.130) and CESD6–GAD4 (difficulty relaxing; *r* = –0.127), whereas in the living-alone group the corresponding edges were weaker (*r* = 0.068 and *r* = –0.070, respectively). These results, which deviate from the total-sample analysis, suggest that emotional tension serves as the central bridging pathway for comorbid depression and anxiety among cohabiting older adults.

Temporal network analysis further revealed dynamic predictive relationships among symptoms over time. Among older adults living alone, the intra-anxiety predictive pattern remained stable, with GAD1 (feeling nervous, anxious, or on edge)–GAD2 (not being able to stop or control worrying) persisting as the central pathway (*r* = 0.392). More importantly, CESD10 (poor sleep quality) consistently predicted subsequent GAD1 (feeling nervous, anxious, or on edge) (cross-cluster edge *r* = 0.160), whereas the corresponding edge in the living-with-family group was smaller (*r* = 0.076). This finding indicates that sleep problems function not only as concurrent bridges but also as stronger longitudinal drivers of increased anxiety among older adults living alone.

In the cohabiting group, GAD1 (feeling nervous, anxious, or on edge)–GAD2 (not being able to stop or control worrying) and GAD5 (being so restless that it is hard to sit still)–GAD6 (becoming easily annoyed or irritable) also constituted the stable backbone of the anxiety network (*r* = 0.443 and *r* = 0.432, respectively). However, cross-symptom effects displayed a different pattern: CESD6 (feeling nervous or afraid)–GAD6 (becoming easily annoyed or irritable) (*r* = 0.128) and CESD6–GAD4 (difficulty relaxing; *r* = –0.125), whereas the corresponding edges in the living-alone group were weaker (*r* = 0.068 and *r* = –0.071, respectively). This indicates that emotional tension serves as the central longitudinal bridging pathway through which depressive symptoms translate into anxiety symptoms among cohabiting older adults.

In summary, the comparison revealed two distinct bridge pathways: the symptom network of older adults living alone was characterized by a “social isolation/loss of cheerfulness–sleep–anxiety” loop, whereas the network of cohabiting older adults followed a “tension–worry–anxiety” sequence. These findings suggest that interventions for solitary older adults should prioritize sleep hygiene and social connectedness to prevent the accumulation of anxiety symptoms over time. For cohabiting older adults, emotion regulation and cognitive restructuring may be more effective in mitigating the cross-network effects of tension and worry.

### 3.4 Network Comparison Test

To further examine the similarities and differences in symptom networks across living arrangements, we conducted the Network Comparison Test (NCT) to systematically evaluate differences in network structure and global strength between the two groups (older adults living alone vs. those living with family).

At the global structure level, the NCT revealed that the difference in network structure between the two groups did not reach conventional statistical significance (structure invariance test: *p* = 0.064), though a marginal trend toward significance was observed. This suggests potential subtle differences in symptom connectivity patterns across living arrangements, warranting cautious interpretation. In contrast, the global strength test indicated no significant difference between groups (strength difference = 0.131, *p* = 0.706), implying that while individual symptom connections may differ, the overall degree of symptom interconnectedness remained stable across the two populations.

Edge-level analyses further identified the specific sources of structural divergence. Significant differences (*p* < 0.05) were primarily found within the depression subnetwork, including CESD2–CESD7 (difficulty concentrating–feeling as happy as one was when younger, *p* = 0.001), CESD4–CESD9 (feeling useless or having difficulty doing things due to aging–feeling unable to carry on with life, *p* = 0.001), and CESD7–CESD10 (feeling as happy as one was when younger–poor sleep quality, *p* = 0.002). These findings suggest nuanced differences in how core depressive symptoms co-occur across living contexts, potentially reflecting distinct emotion processing mechanisms. In addition, specific anxiety symptom connections also differed significantly between groups. For instance, GAD4–GAD6 (difficulty relaxing–becoming easily annoyed or irritable, *p* = 0.002) demonstrated divergent patterns of symptom integration, suggesting variation in anxiety response organization based on living arrangement.

More notably, the NCT identified five cross-cluster (depression–anxiety) edges showing statistically significant differences across groups (*p* < 0.05), including: CESD2–GAD7 (difficulty concentrating–feeling afraid as if something awful might happen, *p* = 0.005), CESD10–GAD6 (poor sleep quality–becoming easily annoyed or irritable, *p* = 0.010), CESD3–GAD1 (feeling depressed or sad–feeling nervous, anxious, or on edge, *p* = 0.018), CESD2–GAD2 (difficulty concentrating–not being able to stop or control worrying, *p* = 0.039), and CESD3–GAD3 (feeling depressed or sad–bothered by trivial matters–excessive worry, *p* = 0.045). These results indicate that in the solitary group, sleep disturbance (CESD10) and attentional difficulties (CESD2) more strongly activated anxiety symptoms. Conversely, in the cohabiting group, emotional tension and irritability served as more prominent “bridge” symptoms between depression and anxiety. (Detailed p-values are presented in **Supplementary Material S3**).

Overall, the NCT results suggest that while global strength remained comparable across groups, meaningful differences existed at the edge level. These distinctions were not only evident within the core depressive symptom network but also extended to cross-cluster bridges between depression and anxiety. The findings underscore the need for tailored interventions based on living arrangement: solitary older adults may benefit more from interventions targeting sleep and attention regulation, whereas those living with family might require emotion-focused strategies to disrupt the transition from emotional tension to anxiety.

## 4 Discussion

### 4.1 Summary of findings

Based on three waves of data from the CLHLS, this study employed propensity score matching (PSM) and network analysis to compare the structure and temporal dynamics of depressive and anxiety symptoms among older adults living alone and those living with family members. The findings revealed a highly interconnected depression–anxiety symptom network in the overall sample, with the strongest cross-symptom association observed between CESD10 (poor sleep quality) and GAD1 (feeling nervous, anxious, or on edge), indicating that sleep disturbances function as a central bridge in comorbid depression and anxiety. This aligns with recent evidence suggesting the centrality of sleep problems in psychiatric disorders (28).

Group-specific results further revealed distinct bridging mechanisms: among older adults living alone, the symptom bridge centered on the “sleep disturbance–anxiety chain,” where CESD10 not only co-occurred with anxiety symptoms but also significantly predicted subsequent anxiety levels. In contrast, older adults living with family exhibited a “tension–worry chain,” with CESD6 (feeling nervous or afraid) serving as a key bridge symptom linking depression and anxiety across time. The Network Comparison Test (NCT) further indicated that while the global strength of the two networks did not significantly differ (*p* = 0.706), marginal differences were observed at the structural level (*p* = 0.064) and in several key edges, particularly cross-symptom connections such as CESD10–GAD6 and CESD2–GAD7. These results provide novel empirical evidence on the symptom transmission mechanisms underlying depression and anxiety in different living arrangements and offer insights for tailored intervention strategies (17, 22).

### 4.2 Theoretical interpretation

The identified “sleep–anxiety chain” (in older adults living alone) and “tension–worry chain” (in those living with family) can be interpreted through the lens of the network theory of mental disorders. Borsboom posited that mental disorders are not caused by latent disease entities but rather emerge from dynamic interactions between symptoms (13). Within this framework, certain symptoms—referred to as bridge symptoms—function as transmission nodes across symptom clusters and, once activated, can initiate cascading effects (15). In this study, CESD10 (poor sleep quality) played a central bridging role in the network of older adults living alone, suggesting that sleep disturbances not only directly reflect depressive states but also facilitate the emergence of anxiety symptoms. This finding is consistent with recent studies emphasizing sleep problems as a bidirectional bridge between depression and anxiety among older adults (29).

In contrast, CESD6 (feeling nervous or afraid) emerged as a cross-symptom and temporal bridge among older adults living with family, supporting the emotion regulation theory, which posits that state-related symptoms such as tension and irritability amplify negative cognitions and worries, thereby exacerbating anxiety responses (30). This pattern is also congruent with the Tripartite Model (31), which highlights negative affect as a shared dimension underlying both depression and anxiety. Subsequent research has suggested that this shared domain may propagate through the “tension–worry” chain in symptom networks (32). The present findings not only validate key assumptions of the model but also provide cross-cultural evidence specific to the Chinese older adult population.

Furthermore, the results reveal the role of loneliness and sleep disturbance within the symptom network of older adults living alone. A growing body of research has shown that loneliness adversely affects sleep quality, which in turn exacerbates symptoms of anxiety and depression (6, 33, 34). Under conditions of social isolation, older adults experience intensified feelings of loneliness, which disrupt the circadian rhythm and lead to a cascading “loneliness–sleep–anxiety” pathway (35–37). In contrast, while older adults living with family may not experience sleep disruption due to social isolation, intensive familial interactions can activate intergenerational conflicts and emotional tension (38, 39). This alternative pathway—”relational stress–emotional tension–anxiety”—suggests that distinct living arrangements give rise to different patterns of symptom transmission. These findings also underscore the capacity of network analysis to detect subtle contextual effects (23).

### 4.3 Practical implications

The findings of this study offer important implications for mental health interventions among older adults. First, for those living alone, sleep disturbances should be prioritized as the primary intervention target. An increasing number of studies have emphasized that improving sleep quality can significantly reduce symptoms of depression and anxiety (40–42). Therefore, mental health interventions for older adults living alone should incorporate sleep management strategies—such as Cognitive Behavioral Therapy for Insomnia (CBT-I) and sleep–wake routine restructuring—alongside programs aimed at enhancing social support to mitigate the cumulative effects of loneliness. Community-based psychological services and digital social support programs may serve as valuable resources to compensate for reduced social connectedness (43).

Second, for older adults living with family, interventions should focus on regulating emotional tension and worry. The symptom network for this group revealed a “tension–worry chain” as a key mechanism, underscoring the importance of mitigating family conflict and emotional reactivity in addressing comorbid anxiety and depression. Evidence suggests that mindfulness-based interventions and emotional regulation skills training can effectively reduce the cross-symptom propagation of tension (44, 45). Furthermore, psychoeducational efforts and intergenerational communication training within families are essential for enabling family members to recognize and alleviate the emotional tension experienced by older adults.

From a public health perspective, this study provides symptom-level evidence for the development of stratified mental health policies. In recent years, the WHO and national aging strategies have highlighted the importance of precision mental health interventions targeting specific symptoms. By identifying core symptoms—such as sleep disturbances and emotional tension—through network analysis, governments and community institutions can tailor interventions based on living arrangements. For example, communities might offer “sleep–loneliness intervention packages” for older adults living alone, while designing “family-based emotional regulation programs” for those living with family. Such symptom-level, network-informed interventions not only enhance precision but also allow for more efficient allocation of limited public health resources (46, 47).

### 4.4 Limitations

Despite the strengths of this study—including a large national sample, longitudinal design, and methodological innovation—several limitations should be acknowledged. First, although the CLHLS is a nationally representative survey, the mental health measures (CES-D-10 and GAD-7) are self-reported and may be subject to social desirability and recall biases (48). Second, while propensity score matching (PSM) was employed to control for demographic differences, unobserved confounders such as personality traits, financial stress, and chronic illness may still have influenced the symptom network structure. Third, although the study utilized three-wave longitudinal data to construct temporal networks, lag-1 modeling does not fully capture causal dynamics; future research should incorporate intensive longitudinal designs or experimental interventions for more robust causal inference (19). Fourth, this study compared only two residential types—living alone and living with family; future studies should include residents of institutional care facilities and other complex living arrangements to provide a more comprehensive understanding of how social contexts shape symptom networks. Finally, although the sample is nationally representative of Chinese older adults, generalizability is largely confined to this context and warrants cross-cultural validation.

## 5 Conclusion

By applying propensity score matching and network analysis, this study offers the first systematic comparison of the structure and dynamics of depression–anxiety symptom networks among older adults living alone versus those living with family in China. While overall network strength was comparable between the two groups, their network structures differed meaningfully: among those living alone, the symptom bridge centered on “sleep disturbance–anxiety,” whereas for those living with family, the core mechanism involved a “tension–worry” chain. These findings support the applicability of the network theory of mental disorders in older Chinese populations and provide empirical justification for symptom-level interventions. Future mental health services and public health strategies should be customized based on living arrangements: for older adults living alone, priority should be given to improving sleep quality and reducing social isolation; for those living with family, interventions should target emotion regulation and family-based support. Such tailored strategies may help alleviate comorbid depression and anxiety more effectively and enhance overall psychological well-being in later life.

## Data Availability

The datasets presented in this study can be found in online repositories. The names of the repository/repositories and accession number(s) can be found below: https://opendata.pku.edu.cn/dataset.xhtml?persistentId=doi:10.18170/DVN/WBO7LK.

## Supplementary material

Supplementary Table 1 Covariate Balance Before and After Propensity Score Matching (PSM) Between Older Adults Living Alone and Those Living with Family.

Supplementary Table 2 95% Credible Intervals for Edge Weights in Contemporaneous and Temporal Networks.

Supplementary Table 3 Top 10 Edges with Smallest P-values from the Network Comparison Test (NCT).

## Appendix A CES-D and GAD-7 Scale Item Descriptions.

**Table:** .

Item Code	Description
CESD1	being bothered by trivial matters
CESD2	difficulty concentrating
CESD3	feeling depressed or sad
CESD4	feeling useless or having difficulty doing things due to aging
CESD5	feeling anxious about the future
CESD6	feeling nervous or afraid
CESD7	feeling as happy as one was when younger
CESD8	feeling lonely
CESD9	feeling unable to carry on with life
CESD10	poor sleep quality
GAD1	feeling nervous, anxious, or on edge
GAD2	not being able to stop or control worrying
GAD3	bothered by trivial matters–excessive worry
GAD4	difficulty relaxing
GAD5	being so restless that it is hard to sit still
GAD6	becoming easily annoyed or irritable
GAD7	feeling afraid as if something awful might happen

Items are based on the CLHLS questionnaire and aligned with the node labels used in the network analysis.
