# Supplementary material for: Bridge symptoms of depression and anxiety among older adults in China: a longitudinal network comparison by living arrangements
Source: Front Psychiatry. 2025 Oct 17;16:1681404. doi: 10.3389/fpsyt.2025.1681404 (PMC12575297; doi:10.3389/fpsyt.2025.1681404)
Supplement: Supplementary Table 1 — Covariate Balance Before and After Propensity Score Matching (PSM) Between Older Adults Living Alone and Those Living with Family. [file Table1.docx]

**Table S1.** Covariate Balance Before and After Propensity Score Matching (PSM) Between Older Adults Living Alone and Those Living with Family

| Covariate | Treat_Before | Control_Before | SMD_Before | Treat_After | Control_After | SMD_After |
| --- | --- | --- | --- | --- | --- | --- |
| distance | 0.36967433778264 | 0.152674035831195 | 1.58948081772745 | 0.36967433778264 | 0.362397778959771 | 0.0532992376692845 |
| age | 84.2333333333333 | 83.0276816608997 | 0.164136858334892 | 84.2333333333333 | 84.0833333333333 | 0.0204209302845629 |
| gender1 | 0.40952380952381 | 0.531141868512111 | -0.247318893 | 0.40952380952381 | 0.419047619047619 | -0.019367338 |
| gender2 | 0.59047619047619 | 0.468858131487889 | 0.247318893250452 | 0.59047619047619 | 0.580952380952381 | 0.0193673377995882 |
| hukou1 | 0.164285714285714 | 0.249134948096886 | -0.228991368 | 0.164285714285714 | 0.157142857142857 | 0.0192771643988747 |
| hukou2 | 0.833333333333333 | 0.750865051903114 | 0.221285619918665 | 0.833333333333333 | 0.842857142857143 | -0.025555063 |
| hukouNA | 0.00238095238095238 | 0 | 0.0488531968746032 | 0.00238095238095238 | 0 | 0.0488531968746032 |
| healthreported1 | 0.15 | 0.118223760092272 | 0.0889912717761746 | 0.15 | 0.133333333333333 | 0.0466760028009337 |
| healthreported2 | 0.345238095238095 | 0.371395617070358 | -0.055016827 | 0.345238095238095 | 0.338095238095238 | 0.015023492575084 |
| healthreported3 | 0.378571428571429 | 0.377162629757785 | 0.00290455397816652 | 0.378571428571429 | 0.397619047619048 | -0.039270929 |
| healthreported4 | 0.111904761904762 | 0.116493656286044 | -0.014556401 | 0.111904761904762 | 0.114285714285714 | -0.007552603 |
| healthreported5 | 0.0142857142857143 | 0.0161476355247982 | -0.015690428 | 0.0142857142857143 | 0.0166666666666667 | -0.020064309 |
| healthreportedNA | 0 | 0.000576701268742791 | -0.026773122 | 0 | 0 | 0 |
| sleephours | 6.86904761904762 | 7.21337946943483 | -0.142402989 | 6.86904761904762 | 6.88809523809524 | -0.007877395 |
| activity1 | 0.0571428571428571 | 0.0818915801614764 | -0.106622503 | 0.0571428571428571 | 0.0547619047619048 | 0.0102576242482777 |
| activity2 | 0.228571428571429 | 0.209919261822376 | 0.0444191719343611 | 0.228571428571429 | 0.24047619047619 | -0.028350576 |
| activity3 | 0.711904761904762 | 0.704728950403691 | 0.0158449923165334 | 0.711904761904762 | 0.704761904761905 | 0.015772225417261 |
| activityNA | 0.00238095238095238 | 0.00346020761245675 | -0.022144529 | 0.00238095238095238 | 0 | 0.0488531968746032 |
| education | 1.49285714285714 | 1.82295271049596 | -0.05892515 | 1.49285714285714 | 1.65 | -0.028051471 |
| hunyin1 | 0.0404761904761905 | 0.556516724336794 | -2.61852137 | 0.0404761904761905 | 0.0404761904761905 | 0 |
| hunyin2 | 0.0285714285714286 | 0.0132641291810842 | 0.0918813065728897 | 0.0285714285714286 | 0.0333333333333333 | -0.028583098 |
| hunyin3 | 0.00952380952380952 | 0.000576701268742791 | 0.0921202953114481 | 0.00952380952380952 | 0.00238095238095238 | 0.073543550676819 |
| hunyin4 | 0.883333333333333 | 0.415801614763552 | 1.45638224404706 | 0.883333333333333 | 0.902380952380952 | -0.059334186 |
| hunyin5 | 0.0333333333333333 | 0.00403690888119954 | 0.163206283380523 | 0.0333333333333333 | 0.0166666666666667 | 0.0928476690885259 |
| hunyinNA | 0.00476190476190476 | 0.00980392156862745 | -0.073240355 | 0.00476190476190476 | 0.00476190476190476 | 0 |
| childmembers | 2.16190476190476 | 2.06113033448674 | 0.039472738539484 | 2.16190476190476 | 2.37380952380952 | -0.083001824 |

**Table S2.** 95% Credible Intervals for Edge Weights in Contemporaneous and Temporal Networks (Full Sample)

| node1 | node2 | mean | ci_lower | ci_upper |
| --- | --- | --- | --- | --- |
| CESD10 | GAD1 | 0.103972982519744 | 0.0352946388679896 | 0.171990621562895 |
| CESD6 | GAD4 | -0.0988503 | -0.169508077 | -0.031039557 |
| CESD6 | GAD6 | 0.0969311384219203 | 0.0311172342502259 | 0.164738077340289 |
| CESD2 | GAD2 | -0.091806504 | -0.157024376 | -0.02359653 |
| CESD7 | GAD6 | -0.091560491 | -0.159352852 | -0.022833847 |
| CESD2 | GAD4 | 0.090498272290819 | 0.0228849019422505 | 0.155891290187308 |
| CESD1 | GAD6 | -0.089892429 | -0.153761205 | -0.020686996 |
| CESD1 | GAD4 | 0.0803226227887297 | 0.00876929038570101 | 0.151424323648918 |
| CESD1 | GAD2 | -0.075948075 | -0.143332967 | -0.00888373 |
| CESD4 | GAD6 | -0.073362183 | -0.143500705 | -0.002654003 |
| CESD1 | GAD3 | -0.071661399 | -0.139362427 | -0.004366675 |
| CESD6 | GAD7 | -0.069830128 | -0.138202193 | -0.000636901 |

**Table S3.** Top 10 Edges with Smallest P-values

| Edge | P_value | Test_statistic |
| --- | --- | --- |
| CESD2 - CESD7 | 0.000999001 | 0.12105731 |
| CESD4 - CESD9 | 0.000999001 | 0.14841619 |
| CESD7 - CESD10 | 0.001998002 | 0.14659436 |
| GAD4 - GAD6 | 0.001998002 | 0.22573907 |
| CESD5 - CESD10 | 0.002997003 | 0.12950456 |
| CESD2 - GAD7 | 0.004995005 | 0.05082831 |
| CESD6 - CESD9 | 0.00999001 | 0.12475712 |
| CESD10 - GAD6 | 0.00999001 | 0.04191887 |
| CESD3 - GAD1 | 0.017982018 | 0.07499899 |
| CESD3 - CESD6 | 0.018981019 | 0.1325158 |
